# The close interaction between hypoxia-related proteins and metastasis in pancarcinomas

**DOI:** 10.1038/s41598-022-15246-y

**Published:** 2022-06-30

**Authors:** Andrés López-Cortés, Lavanya Prathap, Esteban Ortiz-Prado, Nikolaos C. Kyriakidis, Ángela León Cáceres, Isaac Armendáriz-Castillo, Antonella Vera-Guapi, Verónica Yumiceba, Katherine Simbaña-Rivera, Gabriela Echeverría-Garcés, Jennyfer M. García-Cárdenas, Andy Pérez-Villa, Patricia Guevara-Ramírez, Andrea Abad-Sojos, Jhommara Bautista, Lourdes Puig San Andrés, Nelson Varela, Santiago Guerrero

**Affiliations:** 1 Programa de Investigación en Salud Global, Facultad de Ciencias de la Salud, Universidad Internacional SEK, 170302 Quito, Ecuador; 2 Escuela de Medicina, Facultad de Ciencias de la Salud, Universidad de Las Américas, 170124 Quito, Ecuador; 3 Latin American Network for the Implementation and Validation of Clinical Pharmacogenomics Guidelines (RELIVAF-CYTED), 28015 Madrid, Spain; 4 Department of Anatomy, Saveetha Dental College and Hospitals, Saveetha Institute of Medical and Technical Sciences, 600077 Chennai, India; 5 One Health Research Group, Universidad de Las Américas, 170124 Quito, Ecuador; 6 Heidelberg Institute of Global Health, Faculty of Medicine, University of Heidelberg, 69117 Heidelberg, Germany; 7 Instituto Nacional de Investigación en Salud Pública, 170136 Quito, Ecuador; 8 Facultad de Ingenierías y Ciencias Aplicadas, Universidad Internacional SEK, 170302 Quito, Ecuador; 9 Integrated Research and Treatment Center, Center for Sepsis Control and Care (CSCC), Jena University Hospital, 07747 Jena, Germany; 10 Institut Für Humangenetik Lübeck, Universität Zu Lübeck, 23562 Lübeck, Germany; 11 Latin American Network for Cancer Research (LAN-CANCER), Lima, Peru; 12 Laboratorio de Ciencia de Datos Biomédicos, Escuela de Medicina, Facultad de Ciencias Médicas de la Salud y de la Vida, Universidad Internacional del Ecuador, 170113 Quito, Ecuador; 13 BIOscience Research Group, Quito, Ecuador; 14 Laboratory of Chemical Carcinogenesis and Pharmacogenetics, Department of Basic-Clinical Oncology, Faculty of Medicine, University of Chile, 8320000 Santiago, Chile

**Keywords:** Metastasis, Cancer, Molecular medicine

## Abstract

Many primary-tumor subregions exhibit low levels of molecular oxygen and restricted access to nutrients due to poor vascularization in the tissue, phenomenon known as hypoxia. Hypoxic tumors are able to regulate the expression of certain genes and signaling molecules in the microenvironment that shift it towards a more aggressive phenotype. The transcriptional landscape of the tumor favors malignant transformation of neighboring cells and their migration to distant sites. Herein, we focused on identifying key proteins that participate in the signaling crossroads between hypoxic environment and metastasis progression that remain poorly defined. To shed light on these mechanisms, we performed an integrated multi-omics analysis encompassing genomic/transcriptomic alterations of hypoxia-related genes and Buffa hypoxia scores across 17 pancarcinomas taken from the PanCancer Atlas project from The Cancer Genome Atlas consortium, protein–protein interactome network, shortest paths from hypoxia-related proteins to metastatic and angiogenic phenotypes, and drugs involved in current clinical trials to treat the metastatic disease. As results, we identified 30 hypoxia-related proteins highly involved in metastasis and angiogenesis. This set of proteins, validated with the MSK-MET Project, could represent key targets for developing therapies. The upregulation of mRNA was the most prevalent alteration in all cancer types. The highest frequencies of genomic/transcriptomic alterations and hypoxia score belonged to tumor stage 4 and positive metastatic status in all pancarcinomas. The most significantly associated signaling pathways were HIF-1, PI3K-Akt, thyroid hormone, ErbB, FoxO, mTOR, insulin, MAPK, Ras, AMPK, and VEGF. The interactome network revealed high-confidence interactions among hypoxic and metastatic proteins. The analysis of shortest paths revealed several ways to spread metastasis and angiogenesis from hypoxic proteins. Lastly, we identified 23 drugs enrolled in clinical trials focused on metastatic disease treatment. Six of them were involved in advanced-stage clinical trials: aflibercept, bevacizumab, cetuximab, erlotinib, ipatasertib, and panitumumab.

## Introduction

Human cells are highly aerobic and their metabolism rely widely on oxygen supply, matching metabolic demands at the tissue, cellular, and mitochondrial level^1,2^. These highly aerobic cells undergo fundamental shifts in gene expression after sensing changes in the oxygen (O_2_) levels of the microenvironment^3^. These gene expressions are often triggered by metabolic demands, acclimatization or adaptations, causing tissue remodeling, increased cell metabolism, and improved physiological preparedness^4^. In the 90’s, the Hypoxia Inducible Factor (HIF) was identified, purified and cloned. HIF is a transcription factor that regulates oxygen-dependent responses and consists of two protein subunits: HIF-1α and ARNT^3,5,6^. HIF-1α is involved in the protective response towards hypoxia, but also has an important role in oxygen homeostasis, the response to ischemia, pulmonary hypertension, preeclampsia, intrauterine growth retardation, and cancer^7,8^. In 1995, the first full-length clone of the von Hippel-Lindau (VHL) tumor suppressor gene was constructed^9–12^. Subsequently, it was demonstrated in 1999 that VHL targets HIF-1α for oxygen-dependent proteolysis by a proteasomal mechanism^13,14^.

Molecular O_2_ is not only a key nutrient required for aerobic metabolism to maintain intracellular bioenergetics, but it also works as a substrate for several organic and inorganic reactions. Hypoxia occurs in a variety of physiological, as well as, pathological conditions^15^. Half of all solid tumors are characterized by the dynamic gradients of O_2_ distribution and consumption. As result, the tumor environment consist of hypoxic subregions due to changes in tumor metabolism that increase O_2_ demand or insufficient tumor vasculature that decreases O_2_ supply^16–22^. Tumor adaptation to this imbalance between O_2_ demand and supply is associated with increased genomic instability, poor clinical prognosis^19,23^, development of tumor stem cell-protective niches^24,25^, resistance to radio- and chemotherapy^26,27^, and increased proclivity for distal metastases^28–30^.

Metastasis is thought to be the end point of neoplastic cell transformation towards autonomous regulation due to genetic mutations that mediate cell proliferation and invasion^31,32^. In fact, the persistence and lethal relapse of disseminated cancers are driven by stem-like cells that show the ability to regenerate tumors in distal sites^33–36^. Due to treatment pressure by therapeutic resistance, tumor evolution triggers mitochondrial dysfunction and genomic alterations differing substantially between metastatic tumors and primary tumors^37^. Most cancers can be considered curable if the diagnosis occurs before cells are able to spread to secondary tissues; otherwise, cancer is often called incurable as available treatments are not able to prevent metastatic colonization^38–40^. According to Welch and Hurst^32^, the four hallmarks of metastasis are motility and invasion, modulation of the microenvironment, plasticity, and colonization. HIF signaling participates in the metastatic cascade by mediating invasion and migration, intravasation and extravasation, establishment of the premetastatic niche, as well as survival and growth at the distant site^28,41,42^.

Although hypoxia is an adverse and targetable prognostic feature in multiple cancer types^43,44^, an integrated multi-omics analysis between hypoxia and metastasis signaling has not been previously carried out. To shed light on these mechanisms, we evaluated genomic and transcriptomic alterations of 233 hypoxia-related genes (HRGs) and Buffa hypoxia score (HS) per tumor stage and metastatic status across 17 pancarcinomas taken from the PanCancer Atlas (PCA) project from The Cancer Genome Atlas (TCGA) consortium. Additionally, we generated a protein–protein interactome network and tracked the shortest paths from hypoxic proteins to metastatic and angiogenic phenotypes to reveal potential therapeutic targets and drugs for the metastatic disease.

## Results

### OncoPrint of genomic and transcriptomic alterations

**Figure 1:**
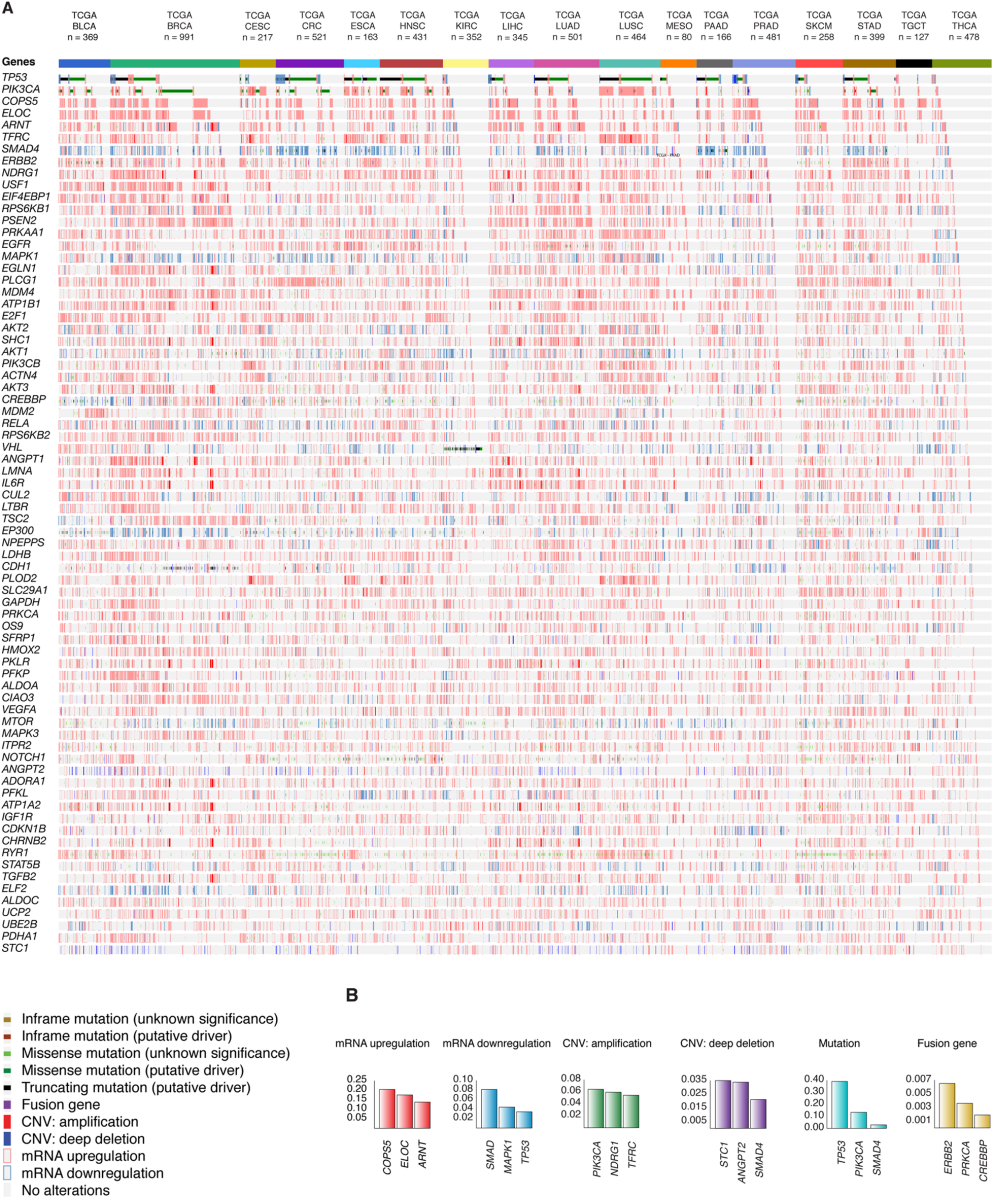
Panoramic landscape of genomic and transcriptomic alterations across pancarcinomas from PCA-TCGA. (**A**) OncoPrint (mRNA high, mRNA low, CNV amplification, CNV deep deletion, putative driver mutation and fusion genes) of the most altered hypoxia-related genes. (**B**) Ranking of the most altered hypoxia-related genes per alteration type. TCGA: The Cancer Genome Atlas; BLCA: bladder urothelial carcinoma; BRCA: breast invasive carcinoma; CESC: cervical squamous cell carcinoma and endocervical carcinoma; CRC: colorectal adenocarcinoma; ESCA: esophageal carcinoma; HNSC: head and neck squamous cell carcinoma; KIRC: kidney renal clear cell carcinoma; LIHC: liver hepatocellular carcinoma; LUAD: lung adenocarcinoma; LUSC: lung squamous cell carcinoma; MESO: mesothelioma; PAAD: pancreatic adenocarcinoma; PRAD: prostate adenocarcinoma; SKCM: skin cutaneous melanoma; STAD: stomach adenocarcinoma; TGCT: testicular germ cell tumors; THCA: thyroid carcinoma; CNV: copy number variant.

We analyzed genomic and transcriptomic alterations of 6343 individuals with 17 pancarcinomas taken from the PCA project from TCGA consortium^45–54^. Figure 1A shows the OncoPrint of 100,643 alterations (mRNA upregulation, mRNA downregulation, copy number variant (CNV) deep deletion, CNV amplification, fusion gene, inframe mutation, truncating mutation, and missense mutation) of the 233 HRGs using the cBioPortal database^55,56^. The PCA-TCGA types were breast invasive carcinoma (BRCA) with 991 (15.6%) individuals, colorectal adenocarcinoma (CRC) with 521 (8.2%) individuals, lung adenocarcinoma (LUAD) with 501 (7.9%) individuals, prostate adenocarcinoma (PRAD) with 481 (7.6%) individuals, thyroid carcinoma (THCA) with 478 (7.5%) individuals, lung squamous cell carcinoma (LUSC) with 464 (7.3%) individuals, head and neck squamous cell carcinoma (HNSC) with 431 (6.8%) individuals, stomach adenocarcinoma (STAD) with 399 (6.3%) individuals, bladder urothelial carcinoma (BLCA) with 369 (5.8%) individuals, kidney renal clear cell carcinoma (KIRC) with 352 (5.5%) individuals, liver hepatocellular carcinoma (LIHC) with 345 (5.4%) individuals, skin cutaneous melanoma (SKCM) with 258 (4.1%) individuals, cervical squamous cell carcinoma and endocervical carcinoma (CESC) with 217 (3.4%) individuals, pancreatic adenocarcinoma (PAAD) with 166 (2.6%) individuals, esophageal carcinoma (ESCA) with 163 (2.6%) individuals, testicular germ cell tumors (TGCT) with 127 (2.0%) individuals, and mesothelioma (MESO) with 80 (1.3%) individuals^57^.

**Figure 2:**
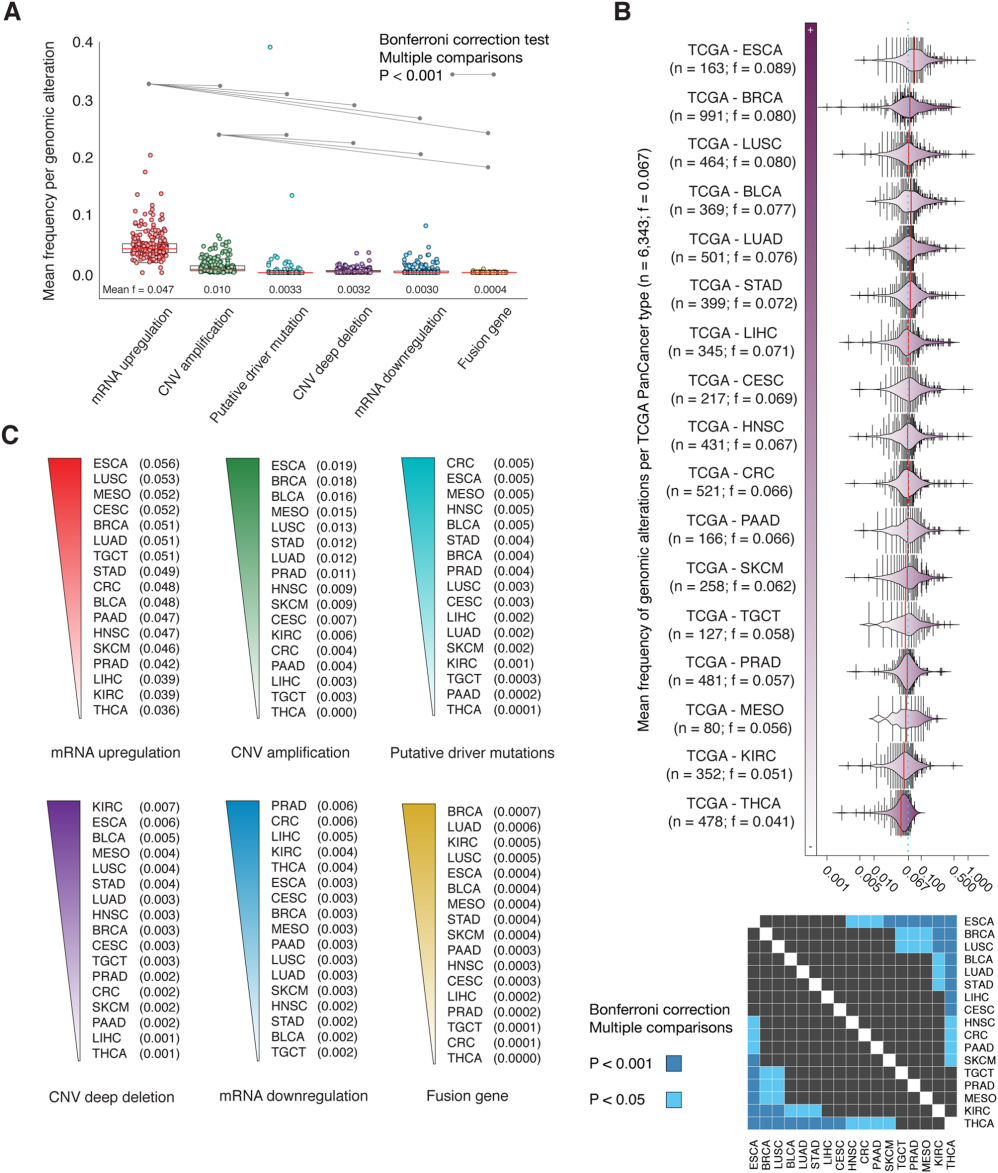
Frequency of genomic and transcriptomic alterations per PCA-TCGA type. (**A**) Mean frequency per alteration type and significant Bonferroni correction (*P* < 0.001) of mRNA upregulation, CNV amplification, putative driver mutation, CNV deep deletion, mRNA downregulation, and fusion gene in comparison with other alterations. (**B**) Ranking of the most altered pancarcinomas from PCA-TCGA according to the mean frequency of alterations, and its validation with a pairwise map of significant Bonferroni correction across PCA-TCGA. (**C**) Ranking of the most altered pancarcinomas per alteration type. TCGA: The Cancer Genome Atlas; BLCA: bladder urothelial carcinoma; BRCA: breast invasive carcinoma; CESC: cervical squamous cell carcinoma and endocervical carcinoma; CRC: colorectal adenocarcinoma; ESCA: esophageal carcinoma; HNSC: head and neck squamous cell carcinoma; KIRC: kidney renal clear cell carcinoma; LIHC: liver hepatocellular carcinoma; LUAD: lung adenocarcinoma; LUSC: lung squamous cell carcinoma; MESO: mesothelioma; PAAD: pancreatic adenocarcinoma; PRAD: prostate adenocarcinoma; SKCM: skin cutaneous melanoma; STAD: stomach adenocarcinoma; TGCT: testicular germ cell tumors; THCA: thyroid carcinoma; CNV: copy number variant.

The genes with the highest mean frequency per genomic/transcriptomic alteration were identified as it follows: mRNA upregulation in *COPS5* (0.204), *ELOC* (0.175), and *ARNT* (0.137); mRNA downregulation in *SMAD4* (0.081), *MAPK1* (0.044), and *TP53* (0.034); CNV amplifications in *PIK3CA* (0.064), *NDRG1* (0.059), and *TFRC* (0.054); CNV deep deletions in *STC1* (0.035), *ANGPT2* (0.034), and *SMAD4* (0.021); putative driver mutations in *TP53* (0.391), *PIK3CA* (0.134), and *SMAD4* (0.029); fusion genes in *ERBB2* (0.006), *PRKCA* (0.004), and *TP53* (0.002); and all genomic alterations in *TP53* (0.476), *PIK3CA* (0.341), and *COPS5* (0.246) (Fig. 1B and Table S1). Additionally, the alteration with the highest mean frequency was mRNA upregulation (0.047), followed by CNV amplification (0.010), putative driver mutation (0.0033), CNV deep deletion (0.0032), mRNA downregulation (0.003), and fusion gene (0.0004). We performed the Bonferroni correction for multiple comparison test to identify statistically significant alterations through the PCA-TCGA. Consequently, we found that mRNA upregulation and CNV amplification were statistically significant altered (*P* < 0.001) across different types of alterations (Fig. 2A).

### Genomic and transcriptomic alterations across pancarcinomas

Figure 2B shows bean plots representing the mean frequency of alterations per pancarcinoma type. The top ten carcinomas with the highest mean frequency of genomic/transcriptomic alterations were ESCA (0.089), BRCA (0.080), LUSC (0.080), BLCA (0.077), LUAD (0.076), STAD (0.072), LIHC (0.071), CESC (0.069), HNSC (0.067), and CRC (0.066) (Table S2 to S18). Additionally, Fig. 2B shows a heatmap of correlation matrix carried out to identify significantly altered carcinomas (*P* < 0.001) by applying the Bonferroni correction test for multiple comparisons. For instance, ESCA, BRCA, and LUSC were significantly altered in comparison to TGTC, PRAD, MESO, KIRC, and THCA. Lastly, carcinomas with the highest mean frequency per alteration were ESCA (0.056), LUSC (0.053), and MESO (0.052) with mRNA upregulation; PRAD (0.006), CRC (0.006), and LIHC (0.005) with mRNA downregulation; ESCA (0.019), BRCA (0.018), and BLCA (0.016) with CNV amplification; KIRC (0.007), ESCA (0.006), and BLCA (0.005) with CNV deep deletion; CRC (0.005), ESCA (0.005), and MESO (0.005) with putative driver mutations; and BRCA (0.0007), LUAD (0.0006), and KIRC (0.0005) with fusion genes (Fig. 2C).

### Tumor stages and metastatic status

**Figure 3:**
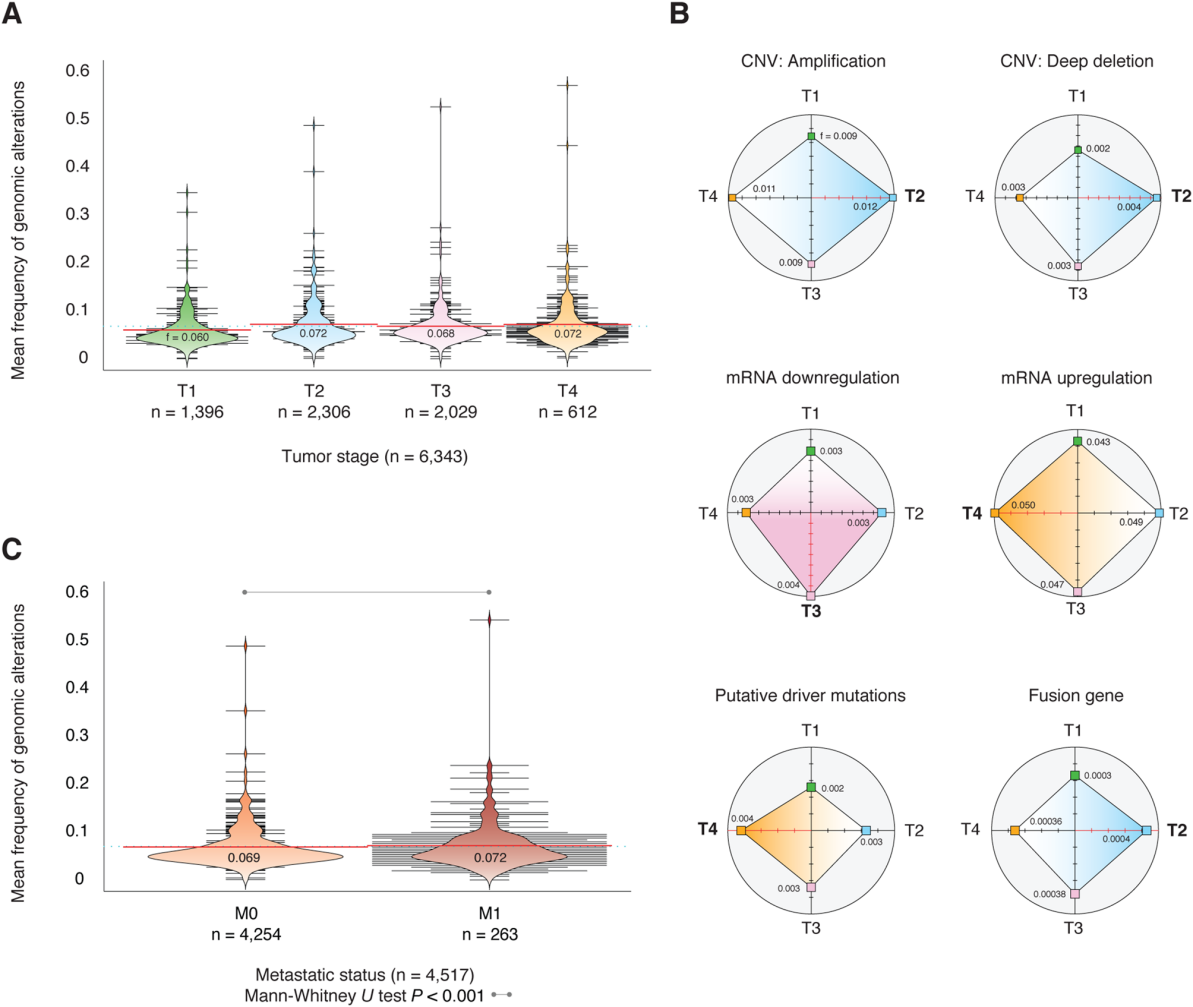
Tumor stages and metastatic status. (**A**) Mean frequency of genomic and transcriptomic alterations per tumor stage across 17 pancarcinomas from PCA-TCGA. (**B**) Mean frequency of each alteration type per tumor stage. (**C**) Mean frequency of genomic and transcriptomic alterations per metastatic status, and its validation through the Mann–Whitney *U* test (*P* < 0.001). CNV: copy number variant.

Figure 3A shows the mean frequency of genomic/transcriptomic alterations across 17 pancarcinomas per tumor stage. The tumor stages with the highest mean frequencies of alterations were T4 (0.072) and T2 (0.072), followed by T3 (0.068) and T1 (0.060) (Table S19 to S22). However, the Bonferroni correction test for multiple comparisons did not show significant differences (*P* > 0.05) between tumor stages. Figure 3B shows the association between alterations and the tumor stage with its highest mean frequency. T2 stage presented the highest mean frequency of CNV amplifications (0.012), CNV deep deletions (0.004), and fusion genes (0.0004); T3 stage presented the highest mean frequency of mRNA downregulation (0.004); and T4 stage presented the highest mean frequency of mRNA upregulation (0.050), and putative driver mutations (0.004). Figure 3C shows the mean frequency of alterations per metastatic status. The mean frequency of M0 status was 0.069 (n = 4254), meanwhile, M1 status was 0.072 (n = 263) (Table S23). Finally, the Mann–Whitney *U* test showed that differences in alterations between M0 and M1 were statistically significant (*P* < 0.001).

### Hypoxia score

**Figure 4:**
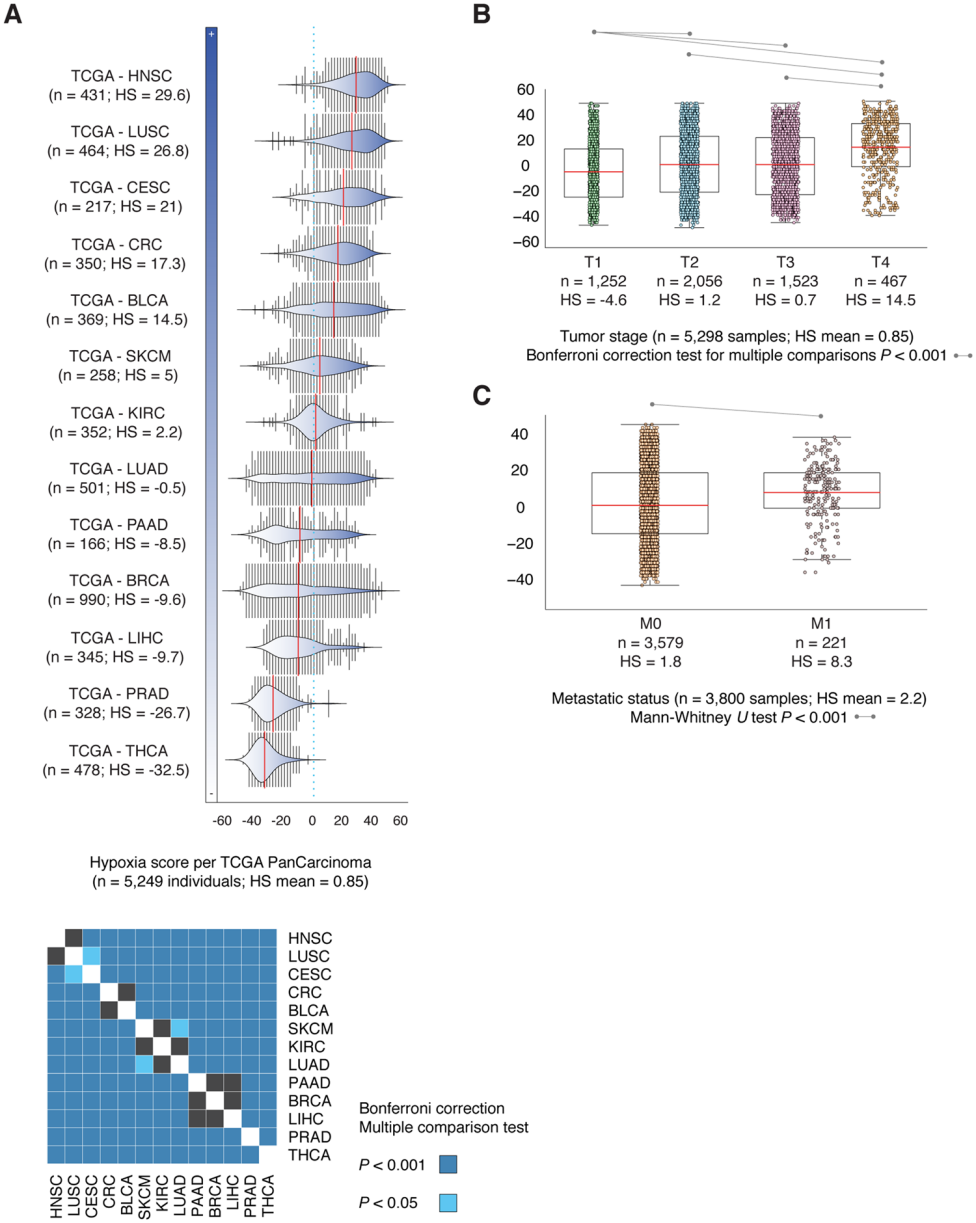
Hypoxia score. (**A**) Hypoxia score mean across 13 PCA-TCGA types, and its validation with a pairwise map of significant Bonferroni correction (*P* < 0.001) across. (**B**) Mean frequency of hypoxia score per tumor stage, and its validation with the Bonferroni correction test. (**C**) Mean frequency of hypoxia score per metastatic status, and its validation with the Mann–Whitney *U* test (*P* < 0.001). TCGA: The Cancer Genome Atlas; BLCA: bladder urothelial carcinoma; BRCA: breast invasive carcinoma; CESC: cervical squamous cell carcinoma and endocervical carcinoma; CRC: colorectal adenocarcinoma; HNSC: head and neck squamous cell carcinoma; KIRC: kidney renal clear cell carcinoma; LIHC: liver hepatocellular carcinoma; LUAD: lung adenocarcinoma; LUSC: lung squamous cell carcinoma; PAAD: pancreatic adenocarcinoma; PRAD: prostate adenocarcinoma; SKCM: skin cutaneous melanoma; THCA: thyroid carcinoma; HS: hypoxia score.

Buffa et al. used information from in vitro experiments combined with in vivo co-expression patterns regarding hypoxia-regulated genes and pathways in order to construct the Buffa hypoxia score^58^. That is, higher mRNA abundance signatures indicate higher levels of hypoxia. Therefore, we quantified tumor hypoxia levels of 5,249 individuals with 13 pancarcinomas using the mentioned score^58^. Figure 4A shows the Buffa HS mean per cancer type. The most hypoxic pancarcinoma was HNSC (29.6), followed by LUSC (26.8), CESC (21), CRC (17.3), BLCA (14.5), SKCM (5), KIRC (2.2), LUAD (-0.5), PAAD (− 8.5), BRCA (− 9.6), LIHC (− 9.7), PRAD (− 26.7), and THCA (− 32.5). Subsequently, we identified highly significant (*P* < 0.001) levels of hypoxia score across 13 pancarcinomas (i.e., HNSC vs THCA) by applying the Bonferroni correction test for multiple comparisons. Figure 4B shows box plots related to the Buffa HS mean per tumor stage (T1-T4) in 5,298 samples of 13 different pancarcinomas. The HS mean in T1 was − 4.6, in T2 was 1.2, in T3 was 0.7, and in T4 was 14.5. Subsequently, we obtained highly significant differences (*P* < 0.001) of HS across tumor stages (i.e., T1 vs T4, T2 vs T4, T3 vs T4) by applying the Bonferroni correction test for multiple comparisons. Finally, Fig. 4C shows box plots of Buffa HS in 3800 metastatic samples. The HS mean of samples without metastasis (M0) was 1.8 and with metastasis (M1) was 8.3. Consequently, we obtained a significant difference (*P* < 0.001) of HS between M0 and M1 through the Bonferroni correction test.

### Protein–protein interactome network

**Figure 5:**
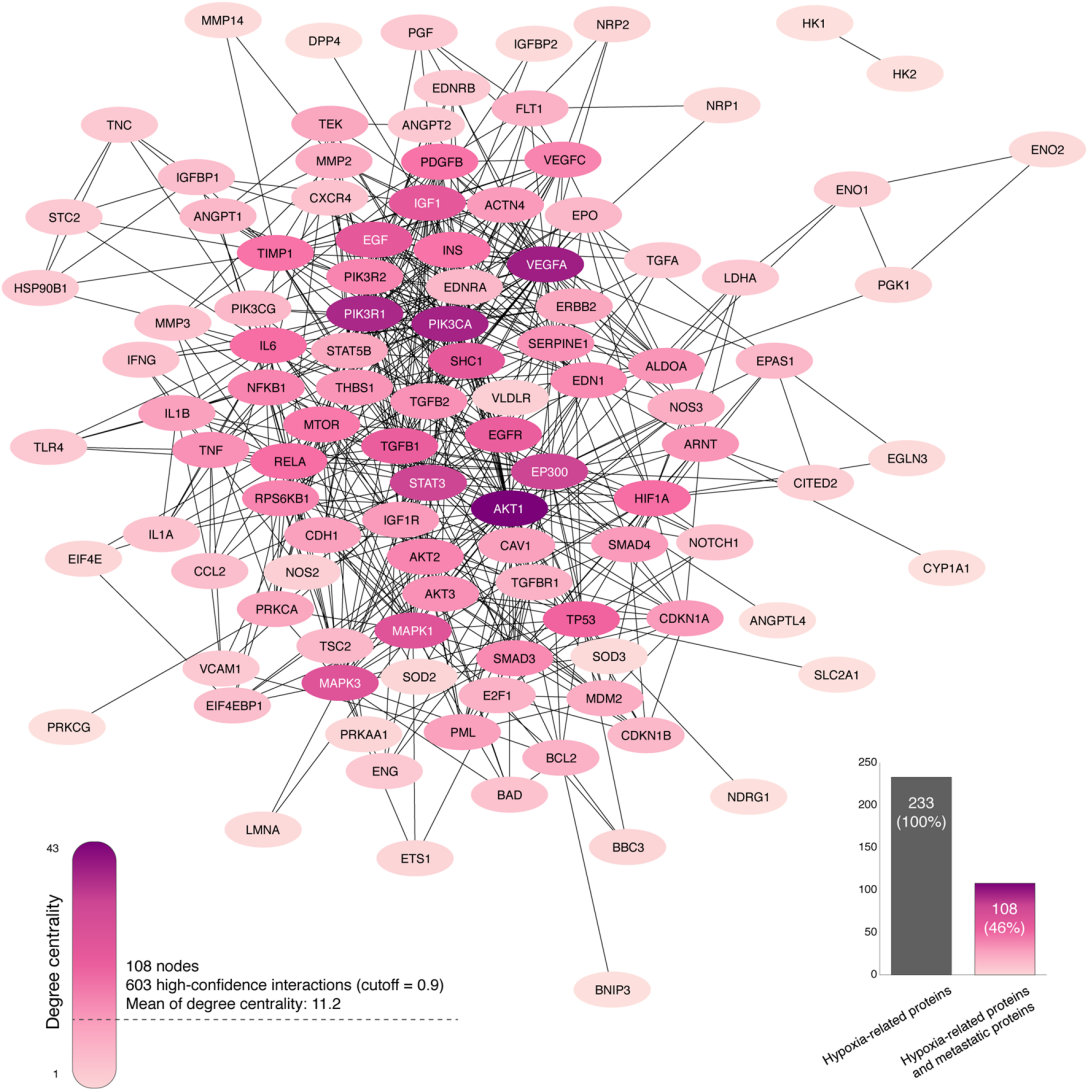
Protein–protein interactome network encompassed by hypoxia-related proteins and metastatic proteins. Network of 108 nodes with at least one high confidence interaction (cutoff = 0.9). Darkest nodes represent proteins with the highest degrees of centrality and a mean of degree of centrality of 11.2.

The PPi network was performed to better understand the connectivity between hypoxia-related proteins and metastatic driver proteins using the String database and the Cytoscape software v.3.7.1^59,60^. Metastatic driver proteins were taken from the Human Cancer Metastasis Database (HCMDB), an integrated database designed to analyze large scale expression data of cancer metastasis^61^. Subsequently, we generated the interactome network encompassing 108 nodes and 603 high-confidence interactions (cutoff = 0.9) (Fig. 5). The mean of degree centrality of the PPi network was 11.2, and the top ten hypoxic proteins involved in metastasis with the highest degree centrality were AKT1 (43), VEGFA (37), PIK3CA (36), PIK3R1 (35), EP300 (29), STAT3 (29), MAPK1 (26), MAPK3 (26), EGF (25), and IGF1 (25) (Tables S24 and S25).

### Shortest paths from hypoxia-related proteins to metastatic and angiogenic phenotypes

**Figure 6:**
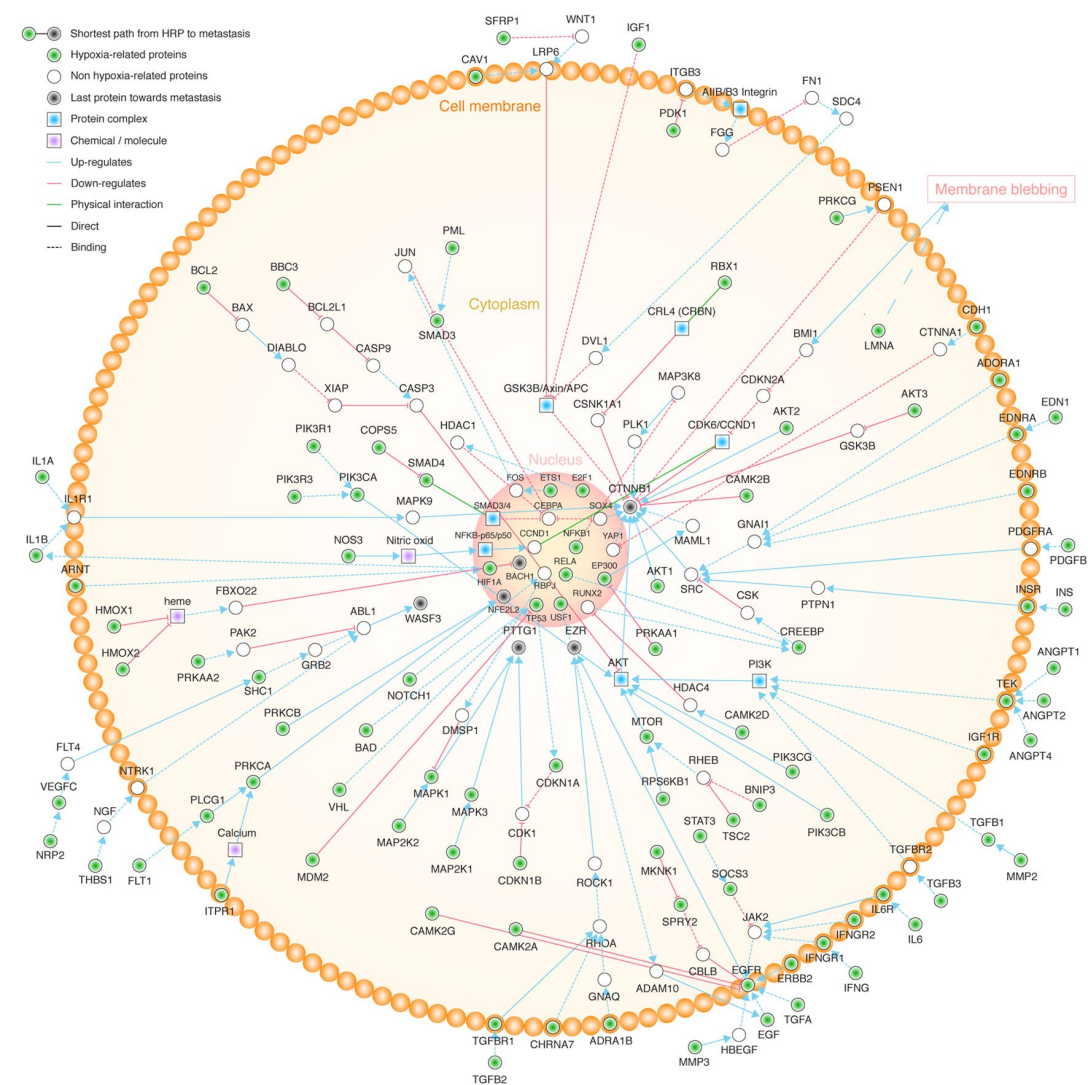
Cell overview of pathways with the shortest distance score from hypoxia-related proteins to the metastatic phenotype. HRP: hypoxia-related proteins.

We analyzed the 233 hypoxia-related proteins by using CancerGeneNet software in order to find the shortest paths to metastatic and angiogenic phenotypes according to Iannuccelli *et al*^62^. On the one hand, we found that 99 (42%) proteins had paths to metastasis, of which, 49 proteins had positive regulation with an average distance of 3.0 and an average path length of 4.0, 16 proteins had negative regulation with an average distance of 3.5 and an average path length of 4.6, and 34 proteins with unknown regulation status had an average distance of 3.2 and an average path length of 4.3. The top ten hypoxia-related proteins with the shortest distance to metastasis were BACH1 (0.8), AKT2 (1.6), AKT1 (1.6), CAMK2B (1.7), EGFR (1.7), MAPK1 (1.7), MAPK3 (1.7), PRKCA (1.7), PRKCB (1.7), and EGF (1.8). The shortest paths from the 99 hypoxia-related proteins to the metastatic phenotype are fully detailed in Fig. 6 and Table S26. On the other hand, angiogenesis, the recruitment of new blood vessels, is an essential component of the metastatic pathway^63^. In our study we found 106 (45%) hypoxic-related proteins with shortest paths to angiogenesis, of which, 73 had positive regulation with an average distance of 3.5 and an average path length of 4.4, 20 proteins had negative regulation with an average distance of 3.9 and an average path length of 4.4, and 13 proteins with unknown regulation status had an average distance of 3.7 and an average path length of 4.7. The top ten hypoxia-related proteins with the shortest distance to angiogenesis were TGFA (0.9), TGFB1 (0.9), TGFB2 (0.9), TGFB3 (0.9), THBS1 (0.9), TIMP1 (0.9), TNF (0.9), VEGFC (0.9), VEGFA (1.2), and MMP2 (1.7) (Table S26).

### Functional enrichment analysis

**Figure 7:**
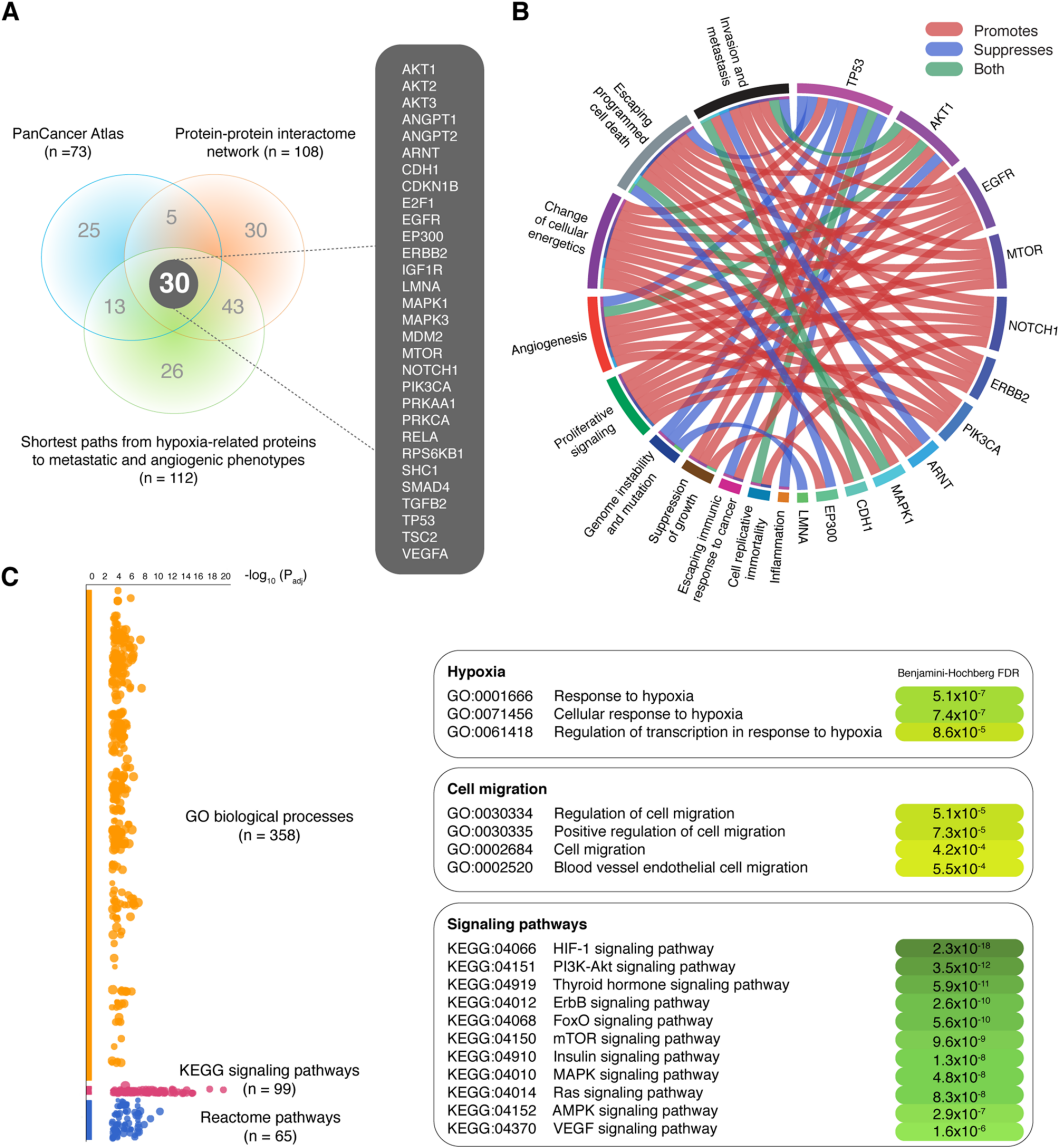
Integration of multi-omics approaches and functional enrichment analysis. (**A**) Venn diagram shows 30 hypoxic/metastatic/angiogenic proteins significantly expressed in the PCA-TCGA, the protein–protein interactome network, and the shortest paths to cancer hallmark phenotypes (metastasis and angiogenesis). (**B**) Circos plot showing that several hypoxia-related proteins promote or suppress cancer hallmark phenotypes. (**C**) Manhattan plot of the functional enrichment analysis showing the most significant GO: biological processes related to hypoxia and cell migration, and the most significant signaling pathways with a Benjamini–Hochberg FDR *q* < 0.001. FDR: false discovery rate; GO: gene ontology; KEGG: Kyoto Encyclopedia of Genes and Genomes.

Figure 7A shows a Venn diagram encompassing the 73 most altered proteins (mean frequency > 0.068) from PCA-TCGA, 108 hypoxic/metastatic proteins with high-confidence interactions (cutoff = 0.9) from the PPi network, and 112 hypoxia-related proteins with the shortest paths to metastatic and angiogenic phenotypes, resulting in 30 essential hypoxia-related proteins highly involved in metastatic signaling (Tables S27 and S28).

The hallmarks of cancer constitute an organizing principle for rationalizing the complexities of neoplastic disease. Nowadays, there are 14 biological capabilities acquired during the multistep development of human tumors^64^. Figure 7B shows a circos plot encompassing 12 of the 30 essential hypoxia-related proteins already characterized as hallmarks of cancer according to the Catalogue of Somatic Mutations in Cancer (COSMIC) Cancer Gene Census (CGC) (https://cancer.sanger.ac.uk/census)^65^. As results, invasion and metastasis were promoted by EGFR, AKT1, MTOR, NOTCH1, ERBB2, MAPK1, and CDH1; escape from programmed cell death was encouraged by AKT1, EGFR, MTOR, NOTCH1, ERBB2, PIK3CA, and MAPK1; changes in cellular energetics were stimulated by TP53, AKT1, EGFR, MTOR, NOTCH1, ERBB2, ARNT, MAPK1, and CDH1; angiogenesis was endorsed by AKT1, EGFR, MTOR, NOTCH1, PIK3CA, and ARNT; proliferative signaling was supported by AKT1, EGFR, MTOR, NOTCH1, ERBB2, and PIK3CA; suppression of growth was promoted by TP53, AKT1, and EP300; escape from immune response during cancer was encouraged by EGFR; lastly, cell replication towards immortality was stimulated by TP53 and NOTCH1.

Subsequently, we performed a functional enrichment analysis of the 30 essential hypoxic/metastatic proteins obtained from the intersection between the PanCancer Atlas, the PPi network, and the shortest paths to metastatic and angiogenic phenotypes analyses. The functional enrichment analysis was performed through the g:Profiler software using the human cancer metastasis proteins as background set. We were able to identify 358 significant gene ontology (GO) biological processes, 99 significant the Kyoto Encyclopedia of Genes and Genomes (KEGG) signaling pathways, and 65 significant Reactome signaling pathways^66–68^. The most significant GO biological processes with Benjamini–Hochberg correction and false discovery rate (FDR) < 0.001 were cellular response to hypoxia and positive regulation of cell migration. The most significant KEGG signaling pathways with Benjamini–Hochberg correction and FDR < 0.001 were HIF-1, PI3K-Akt, thyroid hormone, ErbB, FoxO, mTOR, insulin, MAPK, Ras, AMPK, and VEGF. Lastly, the most significant Reactome signaling pathways with Benjamini–Hochberg correction and FDR < 0.001 were PIP3 activates AKT signaling, PI3K/AKT signaling in cancer, signaling by ERBB2, and MTOR signaling (Fig. 7C and Table S29).

### Validation of the essential hypoxia-related genes involved in metastasis

**Figure 8:**
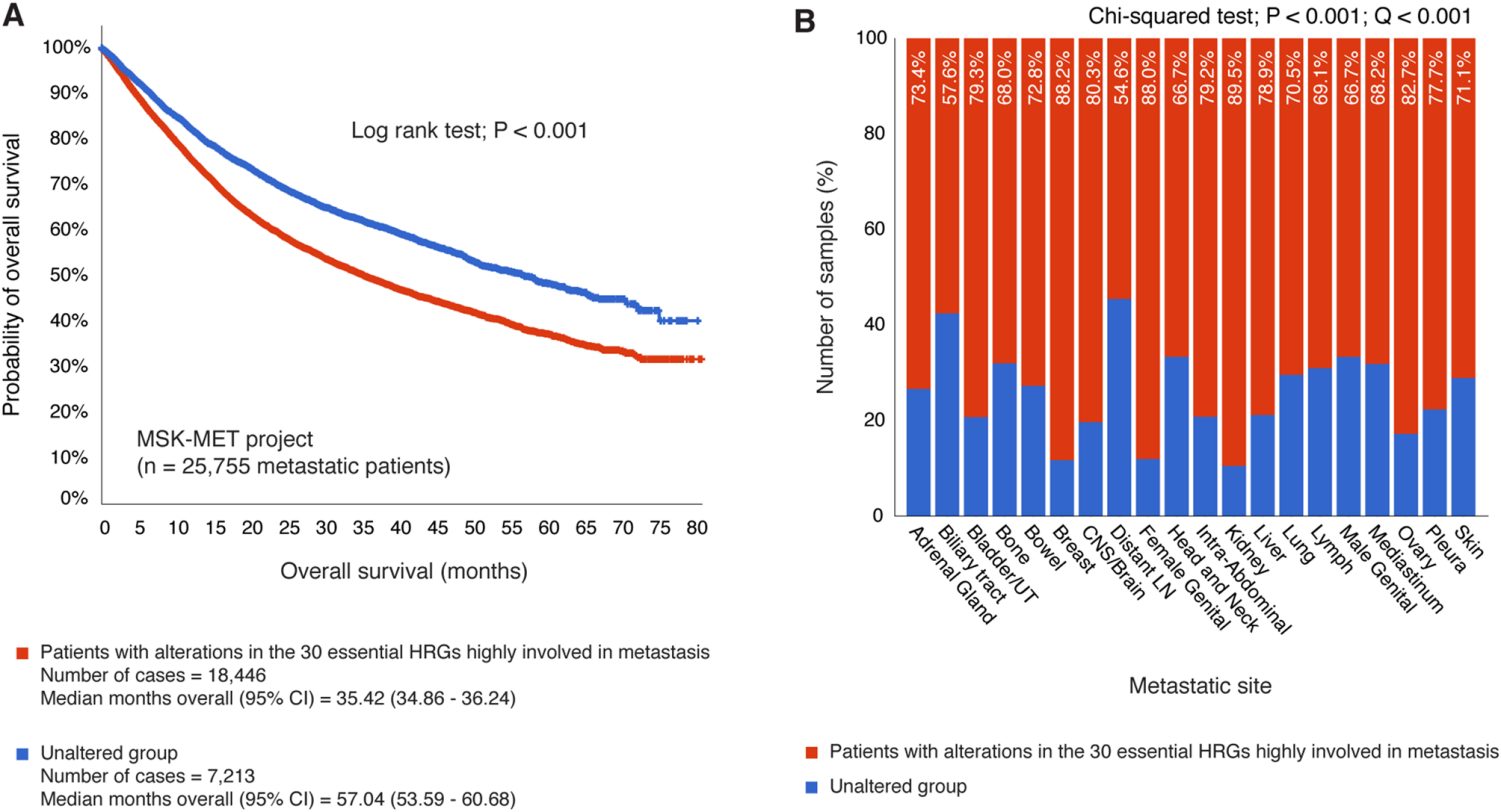
Validation of the essential HRGs through the MSK-MET project. (**A**) Comparison of overall survival between 18,446 patients with alterations in the 30 essential HRGs highly involved in metastasis and 7213 unaltered patients, showing a log rank test *P* < 0.001. (**B**) Percentage of samples with alterations in the 30 essential HRGs and its respective metastatic site. CNS: Central nervous system; LN: lymph node; UT: urothelial; HRG: hypoxia-related genes; CI: coefficient intervals; MSK-MET: Memorial Sloan Kettering—Metastatic Events and Tropisms.

Memorial Sloan Kettering—Metastatic Events and Tropisms (MSK-MET) is an integrated pan-cancer cohort of tumor genomic and clinical outcome data from 25,755 patients with patterns of metastatic dissemination across 50 tumor types^69^. Subsequently, we performed an overall survival analysis comparing patients (n = 18,446) with genomic alterations in the 30 essential hypoxia-related genes versus unaltered patients (n = 7213). As result, the altered group had a median overall survival (95% coefficient intervals) of 35.42 (34.86–36.24) months, and the unaltered group had a median overall survival of 57.04 (53.59–60.68) months. The log rank test showed a statistically significant *P*-value < 0.001 of overall survival (months) between patients with genomic alterations in the 30 essential HRGs and unaltered patients (Fig. 8A).

On the other hand, Fig. 8B shows percentage of samples with alterations in the 30 essential HRGs and its respective metastatic site. Metastasis in kidney had the highest percentage of samples with alterations in the HRGs (89.5%), followed by breast (88.2%), female genital (88.0%), ovary (82.7%), brain (80.3%), bladder (79.3%), intra-abdominal (79.2%), liver (78.9%), pleura (77.7%), adrenal gland (73.4%), bowel (72.8%), skin (71.1%), lung (70.5%), lymph (69.1%), mediastinum (68.2%), bone (68.0%), head and neck (66.7%), male genital (66.7%), biliary tract (57.6%), and distant lymph node (54.6%). The percentage of altered samples in 20 cancer types was statistically significant (*P* < 0.001; *Q* < 0.001) versus the percentage of unaltered samples. Lastly, data can be accessed through the cBioPortal (http://www.cbioportal.org/study/summary?id=msk_met_2021)^69^.

### Drugs involved in clinical trials focused on metastatic disease

**Figure 9:**
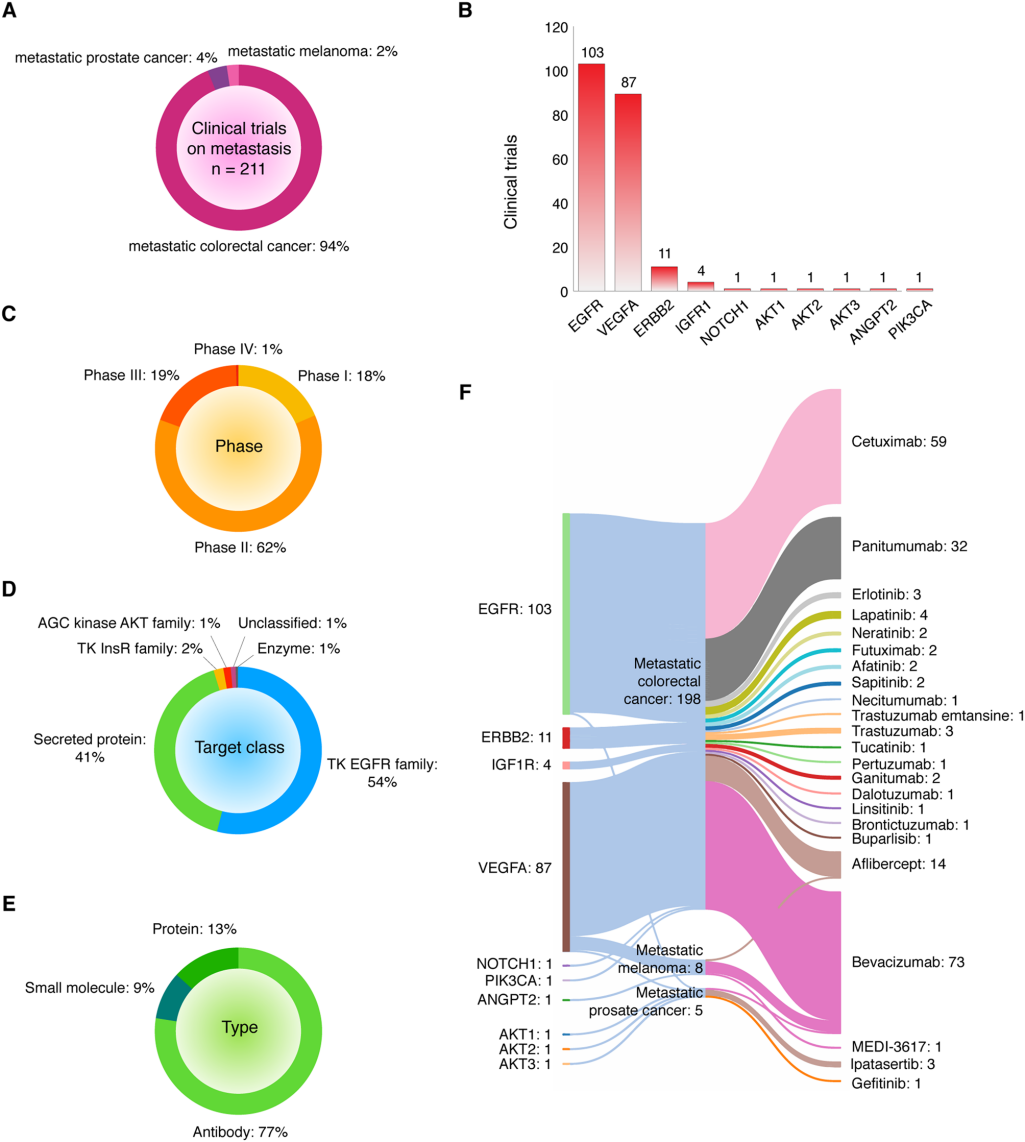
Overview of clinical trials of drugs focused on metastasis. (**A**) Percentage of clinical trials per cancer type. (**B**) Hypoxia-related proteins with highest number of clinical trials on metastasis. (**C**) Phase of clinical trials where drugs are involved. (**D**) Target class. (**E**) Type of drugs. (**F**) Sankey plot showing the therapeutic targets, cancer types, and drugs involved in clinical trials.

Figure 9 shows the current status of drugs in clinical trials for treatment of metastatic disease according to the Open Targets Platform^70^. There are 23 drugs targeting 10 hypoxic/metastatic proteins that have been analyzed in 211 clinical trials. The cancer types with clinical trials focused on metastasis were prostate cancer, colorectal cancer, and melanoma (Fig. 9A). EGFR was the target with the highest percentage of clinical trials in process, recruiting or completed, followed by ERBB2, VEGFA, IGFR1, NOTCH1, AKT1, AKT2, AKT3, ANGPT2, and PIK3CA (Fig. 9B). Most clinical trials were in phase 2 (62%), followed by phase 3 (19%), phase 1 (18%), and phase 4 (1%) (Fig. 9C). Tyrosine kinase EGFR family was the target class with the highest percentage of clinical trials (54%), followed by secreted protein (41%), tyrosine kinase InsR family (2%), AGC kinase AKT family (1%), and enzyme (1%) (Fig. 9D). Antibodies (77%) were the type of drugs focused on metastasis with the highest percentage of clinical trials, followed by proteins (13%) and small molecules (13%) (Fig. 9E). Lastly, Fig. 9F diagrams a Sankey plot comparing the number of clinical trials testing metastatic drugs in different cancer types. Bevacizumab (73), cetuximab (59), panitumumab (32), and aflibercept (14) were the metastatic drugs with the highest number of clinical trials (Table S30).

## Discussion

Hypoxia seems to be a potential micro-environmental factor that induces metastasis through the activation of the HIF signaling pathway. The potential mechanisms evidenced include the reprogramming of metabolism, stem cell phenotype, invasion, vascular facts, suppression of immune system, pre-metastatic niche, intravasation, extravasation, and anti-apoptotic activity^71^. Poor vasculature induces imbalance between pro and anti-angiogenic signals, reducing oxygen delivery into tissues. The poor oxygen delivery produces low oxygen tension in tumor and stromal tissues^72^. In the process of tumor metastasis, invasion is observed to be the first step that is activated by pro-migratory factors induced by mesenchymal stem cells, collagen network formation, and recruitment of macrophages and fibroblasts^64,71^. Hypoxic tumor microenvironment is suggested to aggravate the metastatic initiation through the HIF signaling factor. Hypoxia also triggers the pro-migratory factors and activates the extracellular matrix to support the metastatic process^42^. It is evidenced that the abnormal cancer cell intra and extravasation from the vascular structures is activated by the hypoxic tumor environment and the HIF factor which regulates the cell to cell endothelial adhesion molecules and promotes tumor metastasis^73^.

Hypoxia induces a series of biological changes which contribute to tumorigenesis and the metastatic phenotype^28,74^, both of them are associated with resistance to radio- and chemotherapy, anti-cancer drugs, and immunotherapy. Thus, understanding the molecular signatures of hypoxia is crucial to identify potential therapeutic targets to improve metastatic disease treatments^75^. In this study, we revealed essential hypoxia-related proteins highly involved in metastatic signaling through three approaches that analyze genomic and transcriptomic alterations, protein–protein interactions, and shortest paths from hypoxia to metastatic and angiogenic phenotypes.

The first approach consisted in the analysis of 100,643 genomic/transcriptomic alterations (233 HRGs) belonging to 6343 individuals with 17 pancarcinomas. ESCA, BRCA and LUSC were the most altered cancer types, whereas MESO, KIRC, and THCA were the least altered. The mRNA upregulation was the most significant (Bonferroni correction, *P* < 0.001) genomic alteration since hypoxic tumors overexpress intracellular signals to adapt to the environment^16,76,77^. Overall, the first approach revealed that 73 HRGs presented frequencies of alteration higher than the average (> 0.068). It is important to mention that the 233 hypoxia-related genes were collected from gene ontology terms, signaling pathways, the Buffa signature, and publications on high-altitude adaptive phenotypes. Nevertheless, a limitation of this study is that we did not perform a manual search and curation of publications on individual genes potentially related to hypoxia in order to enrich our initial gene set.

Regarding to genomic/transcriptomic alterations per tumor stage across 17 pancarcinomas, T4 stage presented the highest mean frequencies of mRNA upregulation (0.050) and putative driver mutations (0.004). On the other hand, the mean frequency of genomic alterations in HRGs was significant in patients with metastasis (0.072) in comparison to patients without it (0.069) (Mann–Whitney *U* test, *P* < 0.001), meaning that the number of genomic alterations in the HRGs increases as tumor stage and metastatic status evolves. Smith et al.have proposed this statement through a genetic model in which sequential accumulation of mutations in specific genes (i.e., *ACP*, *KRAS*, *SMAD2*, *SMAD4*, and *TP53*) drives the transition from healthy epithelia to metastatic colorectal cancer^78^.

In order to validate the significant differences in alterations of HRGs found in tumor stages and metastatic status, we analyzed the Buffa hypoxia score across 13 pancarcinomas^58,79^. HNSC, LUSC, and CESC were the most hypoxic cancer types, whereas LIHC, PRAD, and THCA were the least hypoxic, as previously shown by Bhandari *et al*^16^. Regarding to tumor stages, T4 presented the highest HS mean (14.5) with significant differences (Bonferroni correction, *P* < 0.001) between T1 vs T4, T2 vs T4, and T3 vs T4. Regarding to metastatic status, the Buffa HS mean in patients with metastasis (8.3) was significantly higher (Bonferroni correction, *P* < 0.001) than in patients without it (2.2).

The second approach was addressed towards a protein–protein interactome network between hypoxia-related proteins and metastatic driver proteins. In accordance with Li et al., Ivanov et al., and Wurth *et al*^80–82^, protein interactions with therapeutic significance can be revealed by the integration of cancer proteins into networks. Protein interactions regulate essential signals such as proliferation or metastasis, and thus, represent potential targets for drug development and drug discovery. Regarding our networking analysis, we generated an interactome network encompassing 108 nodes and 603 high-confidence edges (cutoff = 0.9) with a mean degree centrality of 11.2. Consequently, the second approach revealed 108 highly connected hypoxic and metastatic proteins.

According to Ianuccelli et al., the value of the third approach permits to bridge the gap between genomic data and cancer phenotypes. However, it should be kept in mind that biology is more complex than graph theory. One important limitation of CancerGenNet is the finding that for up to 20% of the cancer genes we know little about the molecular mechanisms underlying their tumorigenic function^62^. In this context, the third approach was aimed at revealing the shortest paths from HRPs to metastasis^83^. Therefore, we found that 99 hypoxic proteins had paths leading to metastasis from which 49 had positive regulation and 16 had negative regulation. Additionally, the third approach was complemented with the analysis of angiogenic paths because the recruitment of new blood vessels is an essential component of the metastatic pathway^63^. Therefore, we identified 106 hypoxic proteins with shortest paths to the angiogenesis hallmark, of which, 73 had positive regulation and 20 had negative regulation. Then, the compendium of the 112 HRPs with the shortest paths to metastatic and angiogenic phenotypes made up the third approach.

An integrative analysis of the most relevant proteins per approach revealed the 30 essential proteins involved in the signaling crossroads between hypoxia and metastasis: AKT1, AKT2, AKT3, ANGPT1, ANGPT2, ARNT, CDH1, CDKN1B, E2F1, EGFR, EP300, ERBB2, IGF1R, LMNA, MAPK1, MAPK3, MDM2, MTOR, NOTCH1, PIK3CA, PRKAA1, PRKCA, RELA, RPS6KB1, SHC1, SMAD4, TGFB2, TP53, TSC2, and VEGFA.

Subsequently, the functional enrichment analysis of these 30 essential proteins revealed that the most significant GO biological processes (Benjamini–Hochberg correction FDR < 0.001) related to hypoxia were cellular response to hypoxia and regulation of transcription in response to hypoxia; and related to metastasis were regulation of cell migration and blood vessel endothelial cell migration. Additionally, the most significant KEGG pathways (Benjamini–Hochberg correction FDR < 0.001) were HIF-1, PI3K-Akt, thyroid hormone, ErbB, FoxO, mTOR, insulin, MAPK, Ras, AMPK, and VEGF signaling pathways. On the other hand, it is important to mention that several of these proteins not only play a main role in metastasis but also promote other cancer phenotypes. For instance, according to the hallmarks of cancer study^64^ and the COSMIC-CGC database^65^, AKT1, EGFR, ERBB2, MTOR, NOTCH1, and PIK3CA promote the proliferative signaling; AKT1, EP300, and TP53 trigger suppression of growth; EGFR stimulates the escaping immune response to cancer; MTOR and TP53 encourage cell replicative immortality, AKT1, ARNT, EGFR, MTOR, NOTCH1, and PIK3CA promote angiogenesis; AKT1, EGFR, ERBB2, MAPK1, MTOR, NOTCH1, PIK3CA, and TP53 trigger the escaping programmed cell death; and AKT1, ARNT, CDH1, EGFR, ERBB2, MAPK1, MTOR, NOTCH1, and TP53 stimulate the change of cellular energetics (Fig. 7B). Lastly, 16 of these 30 essential proteins were previously catalogued as cancer driver genes according to the Integrative OncoGenomics (IntOGen) database (https://www.intogen.org/search)^84^.

Regarding clinical trials reported on the hypoxic/metastatic essential proteins, the Open Targets Platform is an available resource for the integration of proteogenomics and chemical data to aid systematic drug target prioritization^70^. In this study we identified 23 drugs targeting 10 hypoxic/metastatic proteins that have been analyzed in 211 clinical trials. Six of them (aflibercept, bevacizumab, cetuximab, erlotinib, ipatasertib, and panitumumab) were involved in phases III/IV clinical trials. Ipatasertib, erlotinib, lapatinib, neratinib, afatinib, sapitinib, gefitinib, tucatinib, linsitinib, and buparlisib are small molecules; aflibercept is a recombinant protein; and cetuximab, bevacizumab, panitumumab, futuximab, necitumumab, trastuzumab emtansine, pertuzumab, ganitumab, dalotuzumab, brontictuzumab, and MEDI-3617 are monoclonal antibodies. Lastly, the mechanism of action of these drugs is fully detailed in Table S31.

From a public health perspective, it is fundamental to recognize the holistic view that multi-omics provide to the understanding of cancer; for example, providing accurate selection of patients for the assessment of specific therapies. As reported by Atun et al., the cancer burden continues to grow globally, projecting to have around 24.6 million cases by 2030^85^. Physical health is not the only affected aspect, but millions of people who experience the economic, emotional, and social impacts of the disease and the increasing economic cost to the health systems. Specifically for low-and-middle income countries, there is a gap in the access to health, including a timely quality diagnosis and treatment. Additionally, several studies have highlighted the need to diversify oncological studies to populations representing several ethnic groups along with the development of novel strategies to enhance race/ethnicity data recording and reporting^86–90^. In this sense, multi-omics technologies and public–private investment related to identifying therapeutic targets improving metastatic disease treatments are crucial to reduce inequalities in health and strengthen mechanisms that can improve survival rates of different types of cancer. Relevantly, the effective and ethical use of these technologies would contribute to the development of knowledge and further explanations around the cause of diseases, with the ultimate aim of reducing morbidity and mortality; thus, increasing wellbeing. National health systems should work towards the identification of opportunity costs related to multi-omics research investment and its potential benefits on clinical applications to help diagnose and/or prevent certain diseases. As also stated by Hassin et al., multi-omics approach offers the opportunity to understand the flow of information that underlies disease^91^. The success of the omics approach has to be addressed not only in a technical and/or financial factors-assessment but on the importance of developing conceptual research shifts; focused also on the relations between biological factors and the interrelations with the social determinants of health.

In conclusion, hypoxia and HIF-dependent signaling play an important role in metastasis tumor progression. The hypoxic tumor microenvironment influences both the early and late stages of metastasis. Our findings suggest that individuals with metastasis present higher frequencies of genomic/transcriptomic alterations and Buffa hypoxia score across pancarcinomas from PCA-TCGA. The most altered signaling pathways were HIF-1, PI3K-Akt, thyroid hormone, ErbB, FoxO, mTOR, insulin, MAPK, Ras, AMPK, and VEGF. Finally, since cancer is a group of complex and heterogeneous diseases, the study of multi-omics approaches is an effective way to understand the molecular landscape behind hypoxia and metastasis that revealed 30 potential therapeutic targets and 23 drugs to improve metastatic disease treatments. These drugs can be considered for treating metastasis after being thoroughly evaluated in specific clinical trials.

## Methods

### Gene/protein set

We analyzed a set of 233 HRGs encompassed by the KEGG HIF-1 signaling pathway (n = 109)^92^; the GO terms: response to hypoxia (GO:0001666), cellular response to hypoxia (GO:0071456), and cellular response to decreased oxygen levels (GO:0036293) (n = 102)^66^; the hypoxic genes according to Buffa et al.(n = 52)^58^; and the high-altitude adaptive phenotypes in populations from the Andean Altiplano, Semien Plateau and the Tibetan Plateau according to Bigham (n = 75)^93^.

### OncoPrint of genomic and transcriptomic alterations

Genomic and transcriptomic alterations (mRNA upregulation, mRNA downregulation, CNV deep deletion, CNV amplification, fusion gene, inframe mutation, truncating mutation and missense mutation) were analyzed in 6343 individuals (all with tumor stage) from 17 pancarcinomas from PCA-TCGA (BLCA, BRCA, CESC, CRC, ESCA, HNSC, KIRC, LIHC, LUAD, LUSC, MESO, PAAD, PRAD, SKCM, STAD, TGCT, and THCA). Omics and clinical data related to tumor stage (T1-T4) and metastatic status (M0-M1) were taken from the Genomics Data Commons of the National Cancer Institute (https://portal.gdc.cancer.gov/) and the cBioPortal (http://www.cbioportal.org/)^55,56^. Lastly, the Mann–Whitney *U* test and the Bonferroni correction test for multiple comparisons were performed to determine significant differences (*P* < 0.001) between genomic/transcriptomic alterations, clinical data, and PCA-TCGA type.

### Hypoxia score

The Buffa hypoxia score was analyzed in 5249 tumors from 13 pancarcinomas: HNSC, LUSC, CESC, CRC, BLCA, SKCM, KIRC, LUAD, PAAD, BRCA, LIHC, PRAD, and THCA^58^. An approach for deriving signatures that combine knowledge of gene function and analysis of in vivo co-expression patterns was used to define a common hypoxia signature in cancer. Hypoxia scores were estimated by obtaining the mean expression (log2) of 52 hypoxic genes reported by Buffa *et al*^58,79^. Data related to Buffa hypoxia score, tumor stage, and metastatic status were taken from the Genomics Data Commons and the cBioPortal^55,56^. Lastly, the Bonferroni correction test for multiple comparisons was performed to determine significant differences (*P* < 0.001) between hypoxia score and clinical data.

### Protein–protein interactome network

The PPi network with high-confidence interactions (cutoff = 0.9) and zero node addition was created using the human proteome of Cytoscape StringApp^59,94^, which imports protein–protein interaction data from the String database^59,95–97^. The PPi network was encompassed by hypoxia-related proteins and metastatic driver proteins, which were taken from the Human Cancer Metastasis Database (https://hcmdb.i-sanger.com/). HCMDB is an integrated database designed to analyze large scale expression data of cancer metastasis^61^. The degree centrality of a node represents the number of edges the node has to other nodes in the network and it was calculated using the CytoNCA app^98,99^. All nodes and edges were organized through the organic layout and visualized through the Cytoscape software v.3.7.1^60^. Lastly, the hypoxia-related proteins involved in metastasis signaling were differentiated by colors according to the degree centrality.

### Shortest paths from hypoxia-related proteins to metastatic and angiogenic phenotypes

CancerGeneNet (https://signor.uniroma2.it/CancerGeneNet/) is a resource that links proteins that are frequently mutated in all cancer types to cancer phenotypes. This resource is based on the annotation of experimental information that allows to embed the cancer proteins into the cell network of causal protein relationships curated in SIGNOR^100^. Therefore, this bioinformatics tool allows to infer likely paths of causal interactions linking cancer associated proteins to cancer phenotypes such as metastasis and angiogenesis^62,83,101^. Iannuccelli et al.explained that the shortest paths from a specific protein to cancer phenotypes was programmatically implemented using the shortest path function of *igraph* R package, obtaining a distance score and a path length score^83,102^. Hence, we analyzed the shortest paths from our hypoxic proteins to metastatic and angiogenic phenotypes to better understand the association to these hallmarks of cancer^62,64^.

### Functional enrichment analysis

The enrichment analysis gives scientists curated interpretation of protein sets from omics-scale experiments^57,66,103^. Essential hypoxic and metastatic proteins were analyzed by using g:Profiler version e101_eg48_p14_baf17f0 (https://biit.cs.ut.ee/gprofiler/gost) to obtain significant annotations (Benjamini–Hochberg FDR < 0.001) related to GO biological processes, KEGG signaling pathways, and Reactome signaling pathways^66,68,92^. The functional enrichment analysis was performed using the Human Cancer Metastasis proteins as background set, and annotations were visualized through Manhattan plots. Lastly, significant terms related to hypoxia, metastasis, and oncogenic signaling pathways were manually curated.

### Drugs involved in clinical trials focused on metastatic disease

The Open Targets Platform (https://www.targetvalidation.org) is comprehensive and robust data integration for access to and visualization of potential drug targets associated with several cancer types and metastasis. Additionally, this platform shows all drugs in clinical trials associated with hypoxic/metastatic proteins, detailing its phase, type of drug, action type, and target class^70^.

## Supplementary Information

Supplementary Information.

## Data Availability

All data generated or analyzed during this study are included in this published article (and its Supplementary Information files).

## References

[1] Giaccia AJ, Simon MC, Johnson R (2004). The biology of hypoxia: The role of oxygen sensing in development, normal function, and disease. Genes Dev..

[2] Ortiz-Prado, E., Dunn, J. F., Vasconez, J., Castillo, D. & Viscor, G. Partial pressure of oxygen in the human body: a general review. *Am. J. Blood Res.* (2019). PMC6420699 30899601

[3] Wang GL, Jiang BH, Rue EA, Semenza GL (1995). Hypoxia-inducible factor 1 is a basic-helix-loop-helix-PAS heterodimer regulated by cellular O2 tension. Proc. Natl. Acad. Sci. U. S. A..

[4] Ortiz-Prado E, Natah S, Srinivasan S, Dunn JF (2010). A method for measuring brain partial pressure of oxygen in unanesthetized unrestrained subjects: The effect of acute and chronic hypoxia on brain tissue PO2. J. Neurosci. Methods.

[5] Wang GL, Semenza GL (1993). General involvement of hypoxia-inducible factor 1 in transcriptional response to hypoxia. Proc. Natl. Acad. Sci. U. S. A..

[6] Wang GL, Semenza GL (1995). Purification and characterization of hypoxia-inducible factor. J. Biol. Chem..

[7] Sousa Fialho ML, Abd Jamil AH, Stannard GA, Heather LC (2019). Hypoxia-inducible factor 1 signalling, metabolism and its therapeutic potential in cardiovascular disease. Biochimica et Biophysica Acta Mol. Basis Dis..

[8] Bhattarai D, Xu X, Lee K (2018). Hypoxia-inducible factor-1 (HIF-1) inhibitors from the last decade (2007 to 2016): A “structure–activity relationship” perspective. Med. Res. Rev..

[9] Ohh M (2000). Ubiquitination of hypoxia-inducible factor requires direct binding to the β-domain of the von Hippel-Lindau protein. Nat. Cell Biol..

[10] Lonergan KM (1998). Regulation of hypoxia-inducible mrnas by the Von Hippel-Lindau tumor suppressor protein requires binding to complexes containing elongins B/C and Cul2. Mol. Cell Biol..

[11] Kibel A, Iliopoulos O, DeCaprio JA, Kaelin WG (1995). Binding of the von Hippel-Lindau tumor suppressor protein to Elongin B and C. Science.

[12] Iliopoulos O, Kibel A, Gray S, Kaelin WG (1995). Tumour suppression by the human von hippel-lindau gene product. Nat. Med..

[13] Ivan M (2001). HIFα targeted for VHL-mediated destruction by proline hydroxylation: Implications for O2 sensing. Science.

[14] Maxwell PH (1999). The tumour suppressor protein VHL targets hypoxia-inducible factors for oxygen-dependent proteolysis. Nature.

[15] Xie H, Simon MC (2017). Oxygen availability and metabolic reprogramming in cancer. J. Biol. Chem..

[16] Bhandari V (2019). Molecular landmarks of tumor hypoxia across cancer types. Nat. Genet..

[17] Wilson WR, Hay MP (2011). Targeting hypoxia in cancer therapy. Nat. Rev. Cancer.

[18] Harris AL (2002). Hypoxia—A key regulatory factor in tumour growth. Nat. Rev. Cancer.

[19] Bristow RG, Hill RP (2008). Hypoxia and metabolism: Hypoxia, DNA repair and genetic instability. Nat. Rev. Cancer.

[20] Dhani N, Fyles A, Hedley D, Milosevic M (2015). The clinical significance of hypoxia in human cancers. Semin. Nucl. Med..

[21] Brown JM, Wilson WR (2004). Exploiting tumour hypoxia in cancer treatment. Nat. Rev. Cancer.

[22] Mucaj V, Shay JES, Simon MC (2012). Effects of hypoxia and HIFs on cancer metabolism. Int. J. Hematol..

[23] Luoto KR, Kumareswaran R, Bristow RG (2013). Tumor hypoxia as a driving force in genetic instability. Genome Integrity.

[24] Mohyeldin A, Garzón-Muvdi T, Quiñones-Hinojosa A (2010). Oxygen in stem cell biology: A critical component of the stem cell niche. Cell Stem Cell.

[25] Eliasson P, Jönsson JI (2010). The hematopoietic stem cell niche: Low in oxygen but a nice place to be. J. Cell. Physiol..

[26] Brizel DM, Dodge RK, Clough RW, Dewhirst MW (1999). Oxygenation of head and neck cancer: Changes during radiotherapy and impact on treatment outcome. Radiother. Oncol..

[27] Nordsmark M, Overgaard J (2004). Tumor hypoxia is independent of hemoglobin and prognostic for loco-regional tumor control after primary radiotherapy in advanced head and neck cancer. Acta Oncol..

[28] Rankin EB, Giaccia AJ (2016). Hypoxic control of metastasis. Science.

[29] Gilkes D, Semenza G (2015). Role of hypoxia-inducible factors in breast cancer metastasis. Future Oncol..

[30] Zhong, H. *et al.* Overexpression of hypoxia-inducible factor 1α in common human cancers and their metastases. *Cancer Res.* (1999). 10582706

[31] Ganesh, K. *et al.* L1CAM defines the regenerative origin of metastasis-initiating cells in colorectal cancer. *Nat. Cancer* **1**, (2020). 10.1038/s43018-019-0006-x PMC7351134 32656539

[32] Welch DR, Hurst DR (2019). Defining the Hallmarks of metastasis. Cancer Res..

[33] Batlle E, Clevers H (2017). Cancer stem cells revisited. Nat. Med..

[34] Celià-Terrassa T, Kang Y (2016). Distinctive properties of metastasis-initiating cells. Genes Dev..

[35] Lambert AW, Pattabiraman DR, Weinberg RA (2017). Emerging biological principles of metastasis. Cell.

[36] Massagué J, Obenauf AC (2016). Metastatic colonization by circulating tumour cells. Nature.

[37] López-Cortés A (2020). Prediction of breast cancer proteins involved in immunotherapy, metastasis, and RNA-binding using molecular descriptors and artificial neural networks. Sci. Rep..

[38] Eccles SA, Welch DR (2007). Metastasis: Recent discoveries and novel treatment strategies. Lancet.

[39] Klein CA (2011). Framework models of tumor dormancy from patient-derived observations. Curr. Opin. Genet. Dev..

[40] Steeg PS, Theodorescu D (2008). Metastasis: A therapeutic target for cancer. Nat. Clin. Pract. Oncol..

[41] De Bock K, Mazzone M, Carmeliet P (2011). Antiangiogenic therapy, hypoxia, and metastasis: Risky liaisons, or not?. Nat. Rev. Clin. Oncol..

[42] Semenza GL (2016). The hypoxic tumor microenvironment: A driving force for breast cancer progression. Biochimica et Biophysica Acta Mol. Cell Res..

[43] Janssens GO (2012). Accelerated radiotherapy with carbogen and nicotinamide for laryngeal cancer: Results of a phase III randomized trial. J. Clin. Oncol..

[44] Hoskin PJ, Rojas AM, Bentzen SM, Saunders MI (2010). Radiotherapy with concurrent carbogen and nicotinamide in bladder carcinoma. J. Clin. Oncol..

[45] Hoadley KA (2018). Cell-of-origin patterns dominate the molecular classification of 10,000 tumors from 33 types of cancer. Cell.

[46] Berger AC (2018). A comprehensive pan-cancer molecular study of gynecologic and breast cancers. Cancer Cell.

[47] Liu Y (2018). Comparative molecular analysis of gastrointestinal adenocarcinomas. Cancer Cell.

[48] Campbell JD (2018). Genomic, pathway network, and immunologic features distinguishing squamous carcinomas. Cell Rep..

[49] Ricketts CJ (2018). The cancer genome atlas comprehensive molecular characterization of renal cell carcinoma. Cell Rep..

[50] Huang K (2018). Pathogenic germline variants in 10,389 adult cancers. Cell.

[51] Bailey MH (2018). Comprehensive characterization of cancer driver genes and mutations. Cell.

[52] Gao Q (2018). Driver fusions and their implications in the development and treatment of human cancers. Cell Rep..

[53] Liu J (2018). An integrated TCGA pan-cancer clinical data resource to drive high-quality survival outcome analytics. Cell.

[54] Sanchez-Vega F (2018). Oncogenic signaling pathways in the cancer genome atlas. Cell.

[55] Cerami E (2012). The cBio cancer genomics portal: an open platform for exploring multidimensional cancer genomics data. Cancer Discov..

[56] Gao J (2013). Integrative analysis of complex cancer genomics and clinical profiles using the cBioPortal. Sci. Signal..

[57] Armendáriz-Castillo I (2020). Tcga pan-cancer genomic analysis of alternative lengthening of telomeres (Alt) related genes. Genes.

[58] Buffa FM, Harris AL, West CM, Miller CJ (2010). Large meta-analysis of multiple cancers reveals a common, compact and highly prognostic hypoxia metagene. Br. J. Cancer.

[59] Szklarczyk D (2015). STRING v10: Protein–protein interaction networks, integrated over the tree of life. Nucleic Acids Res..

[60] Shannon P (2003). Cytoscape: A software environment for integrated models of biomolecular interaction networks. Genome Res..

[61] Zheng G (2018). HCMDB: The human cancer metastasis database. Nucleic Acids Res..

[62] Iannuccelli M (2020). CancerGeneNet: Linking driver genes to cancer hallmarks. Nucleic Acids Res..

[63] Zetter BR (1998). Angiogenesis and tumor metastasis. Annu. Rev. Med..

[64] Hanahan D (2022). Hallmarks of cancer: New dimensions. Cancer Discov...

[65] Sondka Z (2018). The COSMIC cancer gene census: Describing genetic dysfunction across all human cancers. Nat. Rev. Cancer.

[66] Raudvere U (2019). g:Profiler: A web server for functional enrichment analysis and conversions of gene lists (2019 update). Nucleic Acids Res..

[67] García-cárdenas JM (2019). Post-transcriptional regulation of colorectal cancer: A focus on RNA-binding proteins. Front. Mol. Sci..

[68] Jassal B (2020). The reactome pathway knowledgebase. Nucleic Acids Res..

[69] Nguyen B (2022). Genomic characterization of metastatic patterns from prospective clinical sequencing of 25,000 patients. Cell.

[70] Carvalho-Silva D (2019). Open targets platform: New developments and updates two years on. Nucleic Acids Res..

[71] Obenauf AC, Massagué J (2015). Surviving at a distance: Organ-specific metastasis. Trends Cancer.

[72] Brown JM, Giaccia AJ (1998). The unique physiology of solid tumors: Opportunities (and problems) for cancer therapy. Cancer Res..

[73] Evans CE, Branco-Price C, Johnson RS (2012). HIF-mediated endothelial response during cancer progression. Int. J. Hematol..

[74] Gilkes DM, Semenza GL (2013). Role of hypoxia-inducible factors in breast cancer metastasis. Future Oncol..

[75] Ye Y (2019). Characterization of hypoxia-associated molecular features to aid hypoxia-targeted therapy. Nat. Metab..

[76] Guerrero S (2020). In silico analyses reveal new putative breast cancer RNA-binding proteins. bioRxiv.

[77] López-Cortés A (2019). OncoOmics approaches to reveal essential genes in breast cancer: A panoramic view from pathogenesis to precision medicine. bioRxiv.

[78] Smith G (2002). Mutations in APC, Kirsten-ras, and p53—Alternative genetic pathways to colorectal cancer. Proc. Natl. Acad. Sci. U. S. A..

[79] Chang WH, Forde D, Lai AG (2019). A novel signature derived from immunoregulatory and hypoxia genes predicts prognosis in liver and five other cancers. J. Transl. Med..

[80] Ivanov AA (2018). The OncoPPi Portal: An integrative resource to explore and prioritize protein-protein interactions for cancer target discovery. Bioinformatics.

[81] Li Z (2017). The OncoPPi network of cancer-focused protein-protein interactions to inform biological insights and therapeutic strategies. Nat. Commun..

[82] Wurth L (2016). UNR/CSDE1 drives a post-transcriptional program to promote melanoma invasion and metastasis. Cancer Cell.

[83] Iannuccelli M (2019). CancerGeneNet: Linking driver genes to cancer hallmarks. Nucleic Acids Res..

[84] Martínez-Jiménez F (2020). A compendium of mutational cancer driver genes. Nat. Rev. Cancer.

[85] Atun R, Knaul FM, Gospodarowicz M (2018). Networks in global cancer—Potential synergies and opportunities. Lancet Global Health.

[86] Guerrero S (2018). Analysis of racial/ethnic representation in select basic and applied cancer research studies. Sci. Rep..

[87] López-Cortés A, Guerrero S, Redal MA, Alvarado AT, Quiñones LA (2017). State of art of cancer pharmacogenomics in Latin American populations. Int. J. Mol. Sci..

[88] Esperón P (2022). Editorial: Pharmacogenetics and pharmacogenomics in Latin America: Ethnic variability, new insights in advances and perspectives: A RELIVAF-CYTED initiative. Front. Pharmacol..

[89] Varela NM (2021). A new insight for the identification of oncogenic variants in breast and prostate cancers in diverse human populations, with a focus on latinos. Front. Pharmacol..

[90] Quinones L (2014). Perception of the usefulness of drug/gene pairs and barriers for pharmacogenomics in Latin America. Curr. Drug Metab..

[91] Hasin Y, Seldin M, Lusis A (2017). Multi-omics approaches to disease. Genome Biol..

[92] Ogata H (1999). KEGG: Kyoto encyclopedia of genes and genomes. Nucleic Acids Res..

[93] Bigham AW (2016). Genetics of human origin and evolution: High-altitude adaptations. Curr. Opin. Genet. Dev..

[94] Doncheva NT, Morris JH, Gorodkin J, Jensen LJ (2019). Cytoscape StringApp: Network analysis and visualization of proteomics data. J. Proteome Res..

[95] López-Cortés A (2018). Gene prioritization, communality analysis, networking and metabolic integrated pathway to better understand breast cancer pathogenesis. Sci. Rep..

[96] López-Cortés A (2019). Pharmacogenomics, biomarker network, and allele frequencies in colorectal cancer. Pharmacogenom. J..

[97] López-Cortés A (2021). In silico analyses of immune system protein interactome network, single-cell RNA sequencing of human tissues, and artificial neural networks reveal potential therapeutic targets for drug repurposing against COVID-19. Front. Pharmacol..

[98] Tang Y, Li M, Wang J, Pan Y, Wu FX (2015). CytoNCA: A cytoscape plugin for centrality analysis and evaluation of protein interaction networks. BioSystems.

[99] López-Cortés A (2022). Pulmonary inflammatory response in lethal COVID-19 reveals potential therapeutic targets and drugs in phases III/IV clinical trials. Front. Pharmacol..

[100] Perfetto L (2016). SIGNOR: A database of causal relationships between biological entities. Nucleic Acids Res..

[101] López-Cortés A (2021). Identification of key proteins in the signaling crossroads between wound healing and cancer hallmark phenotypes. Sci. Rep..

[102] Csardi G, Nepusz T (2006). The igraph software package for complex network research. Int. J. Complex Syst..

[103] Armendáriz-Castillo I (2022). Identification of key proteins from the alternative lengthening of telomeres-associated promyelocytic leukemia nuclear bodies pathway. Biology.

